# Ethylmalonic and Methylsuccinic Acids Disrupt Bioenergetics and Induce Mitochondrial Permeability Transition Through Thiol Redox Modulation in Rat Striatum: Potential Mechanisms Involved in Ethylmalonic Encephalopathy

**DOI:** 10.1007/s11064-026-04801-y

**Published:** 2026-05-27

**Authors:** Manuela Bianchin Marcuzzo, Ângela Beatris Zemniaçak, Jaqueline Santana da Rosa, Bianca Silveira Signorini Verdi, Maria Paula Dalla Vechia Benati, Josyane de Andrade Silveira, Juliana Gomes do Nascimento, Helena de Almeida Moreira, Mateus Dias-Oliveira, Diogo Onofre Souza, Alexandre Umpierrez Amaral, Geancarlo Zanatta, Moacir Wajner, Guilhian Leipnitz

**Affiliations:** 1 Programa de Pós-Graduação em Ciências Biológicas: Bioquímica, Instituto de Ciências Básicas da Saúde, Universidade Federal do Rio Grande do Sul, Porto Alegre, 90035-003 RS Brazil; 2 Postgraduate Programme in Cellular and Molecular Biology (PPGBCM), Center of Biotechnology, Federal University of Rio Grande do Sul, Porto Alegre, 90650-001 RS Brazil; 3 Programa de Pós-Graduação em Atenção Integral à Saúde (UNICRUZ/URI-Erechim/UNIJUÍ), Universidade Regional Integrada do Alto Uruguai e das Missões, Erechim, Rio Grande do Sul Brazil; 4 Serviço de Genética Médica, Hospital de Clínicas de Porto Alegre, Porto Alegre, Brazil; 5 Departamento de Bioquímica, Instituto de Ciências Básicas da Saúde, Universidade Federal do Rio Grande do Sul, Porto Alegre, 90035-003 RS Brazil; 6 Programa de Pós-Graduação em Ciências Biológicas: Neurociências, Instituto de Ciências Básicas da Saúde, Universidade Federal do Rio Grande do Sul, Porto Alegre, 90035-003 RS Brazil; 7 Programa de Pós-Graduação em Ciências Biológicas: Fisiologia, Instituto de Ciências Básicas da Saúde, Universidade Federal do Rio Grande do Sul, Porto Alegre, 90035-003 RS Brazil; 8 Functional Pharmacology and Neuroscience, Department of Surgical Sciences, Uppsala University, Uppsala, 75124 Sweden

**Keywords:** Ethylmalonic encephalopathy, Ethylmalonic acid, Methylsuccinic acid, Bioenergetics, Mitochondrial permeability transition, Striatum

## Abstract

Ethylmalonic (EMA) and methylsuccinic (MSA) acids concentrations are elevated in tissues and body fluids of patients with ethylmalonic encephalopathy (EE), a mitochondrial disorder associated with basal ganglia abnormalities. To clarify the pathophysiology of this disorder, we evaluated the effects of EMA and MSA on bioenergetics, redox homeostasis and mitochondrial permeability transition (MPT) in rat striatum. We verified that EMA and MSA reduced state 3 and uncoupled respiration and inhibited the activities of α-ketoglutarate dehydrogenase and complex IV of the electron transport chain. Both organic acids also decreased mitochondrial membrane potential and Ca^2+^ retention capacity, which were normalized by cyclosporine A and ADP, indicating induction of MPT pore opening. These effects were further mitigated by N-ethylmaleimide and dithiothreitol, suggesting that thiol groups of the MPT pore were oxidized by EMA and MSA. Moreover, EMA and MSA mildly decreased reduced glutathione concentrations, reinforcing that thiol group are oxidized by these organic acids. Therefore, it is presumed that EMA- and MSA-induced bioenergetic impairment associated with MPT pore opening is involved in the pathophysiology of basal ganglia injury observed in EE.

**Supplementary Information:**

The online version contains supplementary material available at 10.1007/s11064-026-04801-y.

## Introduction

Ethylmalonic encephalopathy (EE) is a rare mitochondrial disorder caused by mutations in the *ETHE1* gene, which encodes the enzyme ethylmalonic encephalopathy 1 protein (ETHE1) [1, 2]. ETHE1 is a mitochondrial iron-dependent dioxygenase that plays a crucial role in the degradation of hydrogen sulfide (H₂S). Therefore, the deficient ETHE1 activity results in the accumulation of sulfide and its derivative, thiosulfate, in tissues of the affected individuals [3, 4]. Patients also present elevation of the concentrations of ethylmalonic acid (EMA), derived from the carboxylation of butyryl-CoA, as well as of C4- and C5-acylcarnitine esters, due to the inhibition of short-chain acyl-CoA dehydrogenase (SCAD) by sulfide [3, 5]. Noteworthy, EMA is the most accumulated metabolite and is excreted at high amounts in urine (60–930 mmol/mol of creatinine; normal < 17) [6, 7]. Methylsuccinic acid (MSA), an EMA isomer, is also excreted at higher concentrations, but much lower than EMA (2-200 mmol/mol creatinine; normal < 12) [8–10].

Individuals with EE present with severe neurological manifestations in the first years of life, such as psychomotor regression, generalized infantile hypotonia that evolves to hypertonia, generalized tonic-clonic seizures, progressive spasticity, and spastic tetraparesis. Generalized microvascular damage with petechial purpura, hemorrhagic suffusions of mucosal surfaces, and chronic hemorrhagic diarrhea are also commonly observed [3, 11]. Neuroimaging shows that basal ganglia lesions are a distinctive and common abnormality in EE compared to other brain regions [12, 13]. Other features include abnormalities in dentate nuclei and brainstem, cortical atrophy, as well as periventricular and cerebellar white matter changes that can evolve to diffuse leukoencephalopathy [3, 14, 15]. Brain complex vascular alterations, neuronal loss, gliosis, and augmented vessel density have also been reported [16].

Treatment remains limited and is primarily supportive. It is mainly based on the use of antispastic agents, muscle relaxants and antiepileptic drugs to control neurological manifestations [3, 17]. Another therapeutic option that has shown positive effects on halting disease progression and improving the metabolic abnormalities is the use of the combination of N-acetylcysteine (NAC), a precursor of reduced glutathione (GSH), with the antibiotic metronidazole, a bactericidal agent that decreases gut production of hydrogen sulfide [4, 8, 18]. A low-protein diet restricted in sulfur-containing amino acids has also been associated with metabolic and clinical improvement, particularly when combined with NAC and metronidazole [3, 8]. Finally, patients with severe phenotype have benefited from liver transplantation [17, 19].

Although the pathophysiology of EE is not fully established, mounting evidence has demonstrated that the accumulated metabolites, including EMA, elicit marked toxicity affecting mitochondrial bioenergetics and redox homeostasis [6, 20]. Regarding specifically EMA effects, it was reported that EMA impairs the functioning of the citric acid cycle (CAC) and the electron transport chain (ETC) in rat brain [21, 22]. EMA also inhibits the transport of succinate and malate to the mitochondria and induces mitochondrial permeability transition (MPT) [21, 23, 24]. Furthermore, EMA increases reactive oxygen species (ROS) levels and induces lipid and protein oxidative damage and impairs antioxidant defenses in rat brain [22, 24, 25].

Given that the pathological mechanisms of EE remain poorly understood and that evidence shows that EMA impairs mitochondrial homeostasis, the present study aimed to investigate the in vitro effects of EMA and its structural analogue MSA on mitochondrial bioenergetics, MPT, and redox homeostasis in the striatum of young rats. Mitochondrial calcium (Ca^2+^) retention capacity was also evaluated. Finally, in silico analysis was performed to clarify the mechanisms involved in EMA and MSA effects.

## Materials and Methods

### Reagents

All chemical reagents were purchased from Sigma-Aldrich (St. Louis, MO, USA) unless otherwise stated. EMA (Sigma-Aldrich, USA, Cat. No. 102687) and MSA (Sigma-Aldrich, USA, Cat. No. 81209) solutions were prepared on the day of the experiments, with the pH adjusted to 7.4 using the appropriate buffer for each experimental technique.

### Animals and Ethical Considerations

We used a total of 168 30-day-old male Wistar rats (RRID: RGD_13508588) from the Center for Reproduction and Experimentation of Laboratory Animals (CREAL) at the Federal University of Rio Grande do Sul (UFRGS), Porto Alegre, Brazil. The animals were kept on a 12:12-hour light/dark cycle in a climate-controlled room with controlled temperature (22 ± 1 °C), with free access to water and commercial feed containing 20% (w/w) protein. All experiments were carried out in strict accordance with the Guide for the Care and Use of Laboratory Animals (8th edition, 2011) and approved by the Ethics Committee of UFRGS, Porto Alegre, Brazil (number 47219). Every effort was made to use the smallest possible number of animals without compromising the obtaining of reliable scientific data. Rats were euthanized by decapitation without anesthesia. The striatum was rapidly dissected to prepare homogenates, supernatants, and mitochondrial fractions. The experiments were conducted between 9 a.m. and 4 p.m.

### Preparation of Homogenates, Mitochondrial Fractions and Supernatants

Oxygen consumption was determined in striatal mitochondrial fractions (0.1 mg protein.mL^− 1^) with Oroboros Oxygraph-2k (Innsbruck, Austria) [26]. To measure mitochondrial membrane potential and Ca^2+^ retention capacity, we used mitochondrial fractions (0.75 mg/mL). A mitochondrial fraction (*N* = 1) was obtained from pooling the striatum of four animals. First, the striata were immersed for 10 min in 10 mM HEPES, pH 7.2, containing 225 mM mannitol, 75 mM sucrose, 1 mM EGTA and 0.1% bovine serum albumin (BSA, free of fatty acids). The homogenates were centrifuged at 2,000 x*g* for 5 min to remove cellular debris. The resulting supernatants were centrifuged at 12,000 x*g* for 8 min, and the pellet obtained was resuspended in the same buffer added with 0.04% digitonin to permeabilize synaptosomal membranes. Then, the resultant pellet was resuspended in the original buffer, but without EGTA, and subsequently subjected to a new centrifugation at 12,000 x*g* for 10 min. The final pellet, containing purified mitochondria, was resuspended in 350 µL of EGTA-free culture medium, obtaining an approximate protein concentration of 15 mg.mL^− 1^ [27]. Protein quantification was performed using the Lowry method [28]. Fresh mitochondrial fractions, prepared on the same day as the experiments, were used for the measurement of mitochondrial membrane potential and Ca^2+^ retention capacity.

For oxidative stress experiments, the striatum was dissected and homogenized (1:10 w/v) in 20 mM sodium phosphate buffer, pH 7.4, containing 140 mM KCl. After centrifugation at 750 x*g* for 10 min (4 °C), the supernatants, which consisted of a suspension of mixed and preserved organelles, including mitochondria [29], were used for the evaluation of redox homeostasis parameters. Each supernatant (*N* = 1) was obtained from the striatum of one rat. The supernatants were incubated in the absence (control group) or presence of EMA or MSA (1–5 mM) for 1 h at 37 °C. After incubation, aliquots were separated for the measurement of GSH levels, as well as glutathione peroxidase (GPx) and superoxide dismutase (SOD) activities. In another experiment, mitochondrial fractions were incubated in the absence (control group) or presence of EMA or MSA (5 mM) for 1 h at 37 °C and then used to measure GSH levels.

For the investigation of energy metabolism parameters, the striatum was homogenized (1:10 w/v) in SET buffer (250 mM sucrose, 2.0 mM EDTA and 10 mM Trizma base), pH 7.4. The homogenates were centrifuged at 800 x*g* for 10 min at 4 °C and the supernatants obtained were used for evaluating the activities of CAC enzymes and ETC complexes. Each supernatant (*N* = 1) was obtained from the striatum of one rat. Samples were incubated at 37 °C for 30 min, under controlled conditions, together with reagents specific to each assay and EMA or MSA (1–5 mM). This step aimed to allow enzymatic activation and the occurrence of biochemical reactions necessary for quantifying the metabolic parameters.

### Parameters

#### Mitochondrial Respiration

Oxygen consumption (0.1 mg protein.mL^− 1^) supported by pyruvate (5 mM), malate (0.5 mM), and glutamate (10 mM) (PMG) plus succinate (10 mM) was measured in an Oroboros Oxygraph-2k at 37 °C. Mitochondrial respiration rates in states 3 and 4, uncoupled respiration, as well as the respiratory control ratio (RCR, ratio between states 3 and 4) were determined. EMA or MSA (1–5 mM) was added to the reaction medium at the beginning of the experiment. State 3 was induced by the addition of ADP (1 mM), while state 4 was obtained by the addition of oligomycin A (1 µg.mL^− 1^). Uncoupled respiration was assessed by adding the uncoupler CCCP (carbonyl cyanide 3-chlorophenyl hydrazone; two pulses of 0.5 µM) to induce the maximum rate of oxygen consumption [30]. Data were expressed as pmol O_2_.s^− 1^.mg protein^− 1^.

#### Activities of Citric Acid Cycle (CAC) Enzymes

Citrate synthase (CS) activity was determined by spectrophotometric detection of 5,5′-dithiobis(2-nitrobenzoic acid) (DTNB) reduction [31]. The assay was performed at 37 °C using samples with approximately 2 µg of protein in a medium containing 5 mM potassium phosphate, pH 7.4, 300 mM sucrose, 1 mM EGTA, 0.1% bovine serum albumin (BSA), 5 mM MOPS, 0.1% Triton X-100, 0.1 mM DTNB, 0.1 mM acetyl-CoA, and 0.2 mM oxaloacetate. The reading of 5-thio-2-nitrobenzoic acid (TNB) was performed at 412 nm.

Malate dehydrogenase (MDH) activity was assessed as described by Kitto [32], by monitoring the decrease in NADH fluorescence at 37 °C. The reaction was carried out with approximately 1 µg of protein, 50 mM potassium phosphate, pH 7.4, 10 µM rotenone, 0.1% Triton X-100, 0.14 mM NADH, and 0.3 mM oxaloacetate. The fluorescence was read at an excitation wavelength of 340 nm and an emission wavelength of 450 nm.

The activity of α-ketoglutarate dehydrogenase (α-KGDH) was determined by monitoring NADH fluorescence at an excitation wavelength of 340 nm and an emission wavelength of 450 nm [33]. The assay was performed at 37 °C in a medium containing sample with approximately 20 µg of protein, 0.2 mM thiamine pyrophosphate, 2 mM NAD^+^, 1 mM MgCl_2_, 0.4 mM ADP, 10 µM rotenone, 0.1% (v/v) Triton X-100, 50 mM potassium phosphate buffer, pH 7.4, and 0.2 mM EGTA. The reaction was initiated by the addition of 0.12 mM HS-CoA and 1 mM α-ketoglutarate.

Succinate dehydrogenase (SDH) activity was determined as described by Fischer et al. [34], monitoring the reduction of dichlorophenolindophenol (DCIP). The assay was performed at 25 °C in a medium containing approximately 30 µg of protein, 40 mM potassium phosphate, pH 7.4, 16 mM sodium succinate, 4 mM sodium azide, 7 µM rotenone, 8 µM DCIP and 1 mM phenazine methosulfate. The absorbance was read at 600 nm.

All these parameters were measured using a SpectraMax M5 microplate reader (San Jose, CA, USA). The activities were calculated as µmol/min.mg protein or nmol.min^− 1^.mg protein^− 1^.

#### Activities of Electron Transport Chain (ETC) Complexes

NADH dehydrogenase activity (complex I) was measured according to the protocol described by Michelini et al. (2014). The activities of succinate-2,6-dichloroindophenol (DCIP)-oxidoreductase (complex II) and succinate: cytochrome *c* oxidoreductase (complex II-III) were determined using the method originally described by Fischer and colleagues [34]. Cytochrome *c* oxidase activity (complex IV) was determined according to Rustin et al. [35]. Absorbance changes were recorded on a SpectraMax M5 plate reader (Molecular Devices, Sunnyvale, CA, USA), and the results were expressed as nmol.min^− 1^.mg protein^− 1^. The activities were calculated as nmol.min^− 1^.mg protein^− 1^.

#### Structural Data

MSA, EMA, lipoic acid (LA) and succinate chemical structures were obtained from PubChem server [36]. NAD+, FAD, enamine-ThDP and CoA-SH were obtained from crystallographic structures. Prior to use, all structures underwent protonation analysis at pH 7.4 and energy minimization using the MarvinSketch code (ChemAxon Marvin Suite version 20.20.0) or Avogadro software v 1.2.0 [37].

Target proteins were modelled as described in a previous study [24]. E1 and E3 subunits of α-KGDH were modelled using template structures 2Y0P, 6H05 and 6I4T retrieved from the Protein Data Bank (https://www.rcsb.org/) [38], and amino acid sequences F1SSH8, P09623 and P09623 retrieved from the Uniprot server (www.uniprot.org) [39]. The template structure PDBId 2LCK and the amino acid sequence Q9UBX3 and Q02978 were used to model the mitochondrial dicarboxylate carrier and the mitochondrial α-ketoglutarate/malate carrier, respectively. Sequence Q9H936 and template structure PDBId 1OKC were employed to model the mitochondrial glutamate carrier. The ProPKA algorithm in the PDB2PQR Server Protonation was employed to protonate all models at pH 7.4 [40]. Previous equilibrated structures of the mitochondrial carriers into a 1-palmitoyl-2-oleoyl-sn-glycero-3-phosphocholine (POPC) lipid bilayer were employed during docking simulations.

#### Molecular Simulations

Molecular docking simulations were performed with the Vina algorithm [41] using the modeled structures for α-KGDH, mitochondrial dicarboxylate carrier, mitochondrial glutamate carrier, and mitochondrial α-ketoglutarate/ malate carrier. The grid box was created based on the size of the crystallographic ligands for each binding site analyzed. For every binding site assessed in α-KGDH, docking was repeated until the generation of 100 poses per ligand. For the membrane mitochondrial carriers, the ensemble docking method was employed using 16, 17, and 22 distinct conformations of dicarboxylate, α-ketoglutarate/ malate and glutamate carriers, respectively. Two rounds of docking were performed for each ligand. In the first round, the grid box was created based on the size of the pore in each analyzed carrier. In the second, the grid box was created based on the size of the binding site identified for any ligand during round 1. For each carrier conformation, docking was repeated 20 times (~ 100 poses). Best representative poses in α-KGDH and mitochondrial carriers were selected based on the calculated binding energy, and figures were built with Pymol (The PyMOL Molecular Graphics System, Version 2.3.2, Schrödinger, LLC) and Inkscape (https://inkscape.org/) softwares.

#### Mitochondrial Membrane Potential

Mitochondrial membrane potential (ΔΨm) was assessed by monitoring the fluorescence of the cationic dye safranin O (5 µM) using a Hitachi F-2500 spectrofluorometer (Tokyo, Japan), with excitation and emission wavelengths set at 495 nm and 586 nm, respectively [42]. Fluorescence data were quantified by calculating the variation in fluorescence arbitrary units (FAU) between 150 and 250 s from the start of the assay.

#### Mitochondrial Ca^2+^ Retention Capacity

Mitochondrial Ca²⁺ retention capacity was determined by real-time monitoring of free extramitochondrial Ca²⁺ levels using the fluorescent indicator Calcium Green-5 N (0.2 µM). Experiments were conducted on a Hitachi F-2500 spectrofluorometer (Tokyo, Japan), operating at an excitation wavelength of 506 nm and emission wavelength of 532 nm, as previously described [43].

#### Reduced Glutathione (GSH) Levels

The concentrations of GSH were quantified according to the method described by Browne and Armstrong [44]. The assay is based on the reaction between GSH and o-phthaldialdehyde, which results in the formation of a fluorescent compound. Fluorescence was measured with excitation at 350 nm and emission at 420 nm. The results were expressed as nmol of GSH.mg of protein^− 1^.

#### Activities of Antioxidant Enzymes

The activities of glutathione peroxidase (GPx) and superoxide dismutase (SOD) were determined based on the absorbance read on a SpectraMax M5 plate reader (Molecular Devices, CA, USA). The specific activities were calculated as U.mg protein^− 1^.

GPx activity was determined as previously described [45], monitoring the decrease in NADPH absorbance at 340 nm in a reaction medium containing 600 µL of 100 mM potassium phosphate buffer, pH 7.0, supplemented with 1 mM EDTA, 0.4 mM sodium azide, 2 mM reduced GSH, 0.1 U/mL glutathione reductase, 0.5 mM tert-butylhydroperoxide, 0.1 mM NADPH and samples containing approximately 3 µg of protein.

SOD activity was determined as described by Marklund [46]. The assay was conducted in a medium containing 50 mM Tris-HCl, 1 mM EDTA, pH 8.2, 80 U/mL catalase, 0.38 mM pyrogallol, and samples containing approximately 1 µg of protein. The rate of pyrogallol auto-oxidation was monitored by spectrophotometry, with absorbance readings at 420 nm. SOD activity was quantified based on a standard curve obtained from purified SOD.

### Statistical Analysis

The mean ± standard deviation (SD) is shown in the figures. For all experiments, group means were calculated using independent biological samples, consisting of either supernatants (each derived from the striatum of a single rat) or mitochondrial fractions (each obtained by pooling striatal tissue from four rats). Data were analyzed using the SPSS software (New York, NY, USA). Normality was analyzed by the Shapiro-Wilk test. One-way ANOVA followed by the post hoc Duncan multiple range test was used when F was significant. Differences were significant when *P* < 0.05. The sample size was determined using Minitab software for an experiment with four groups, resulting in a required N of 4. We assumed a target power of 0.8, an SD of 10%, and a difference of 30%. These data were assumed in accordance with previous studies from our group [23, 47]. However, in some experiments, we used N slightly different than the calculated value with Minitab software, but in accordance with previous works [23, 47].

## Results

### EMA Decreases Mitochondrial Respiration in Striatal Mitochondrial Fractions

**Fig. 1:**
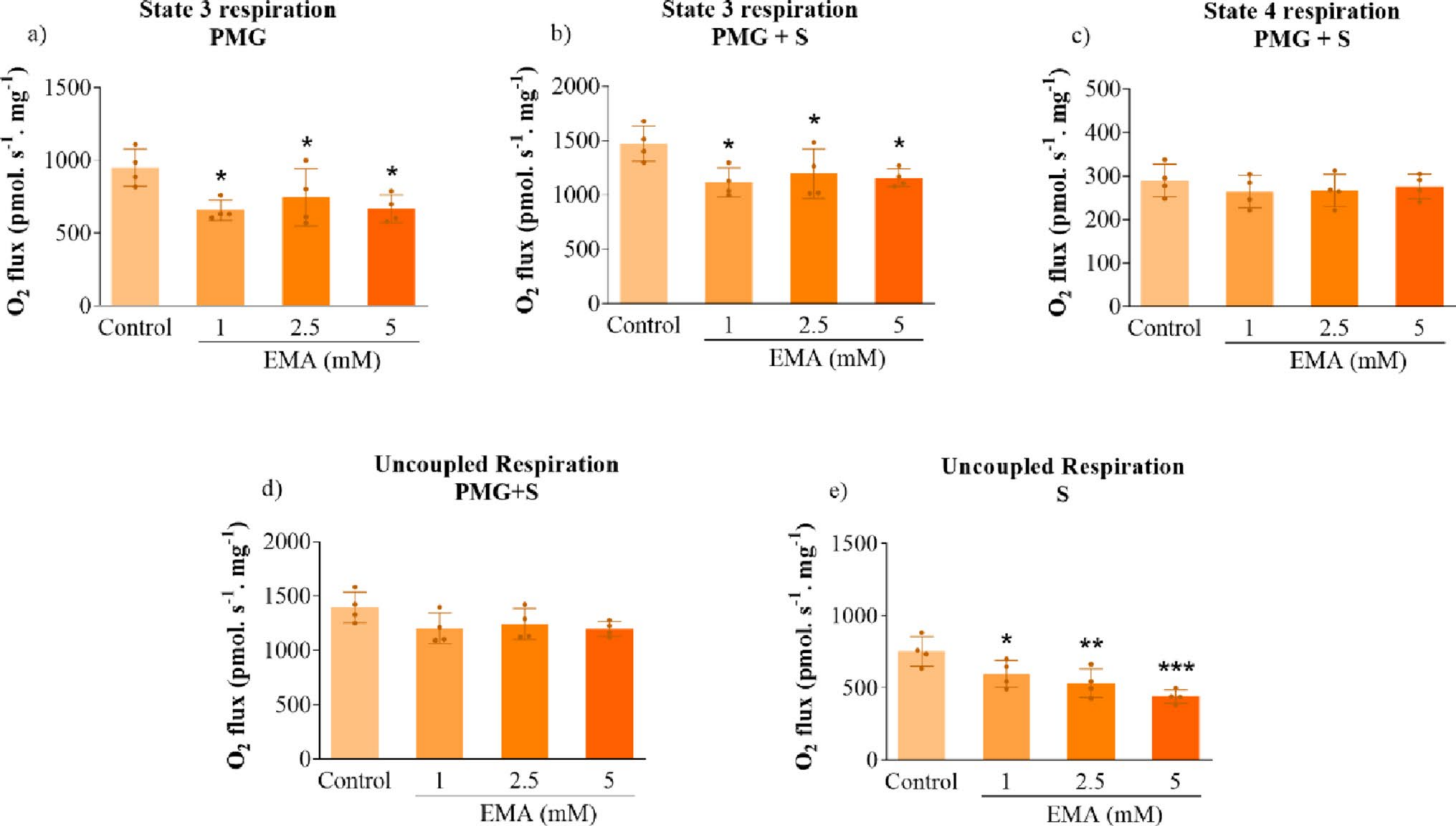
Effects of ethylmalonic acid (EMA) (1–5 mM) on mitochondrial respiration in rat striatum. State 3 respiration was measured in the presence of ADP (1 mM), and pyruvate (5 mM), malate (0.5 mM), and glutamate (10 mM) (PMG) (**a**) plus succinate (S; 10 mM) (**b**) as substrates to assess respiration linked to oxidative phosphorylation; state 4 was stimulated by oligomycin (ATP synthase inhibitor; 1 µg.mL^− 1^), to evaluate mitochondrial respiration not linked to ATP formation (resting respiration) (**c**). Uncoupled respiration was stimulated by CCCP (uncoupler; two pulses of 0.5 µM) (**d** and **e**). Values are mean ± standard deviation (*N* = 4). *P < 0.05, **P < 0.01, ***P < 0.001, compared to the control group (Duncan’s multiple range test)

**Fig. 2:**
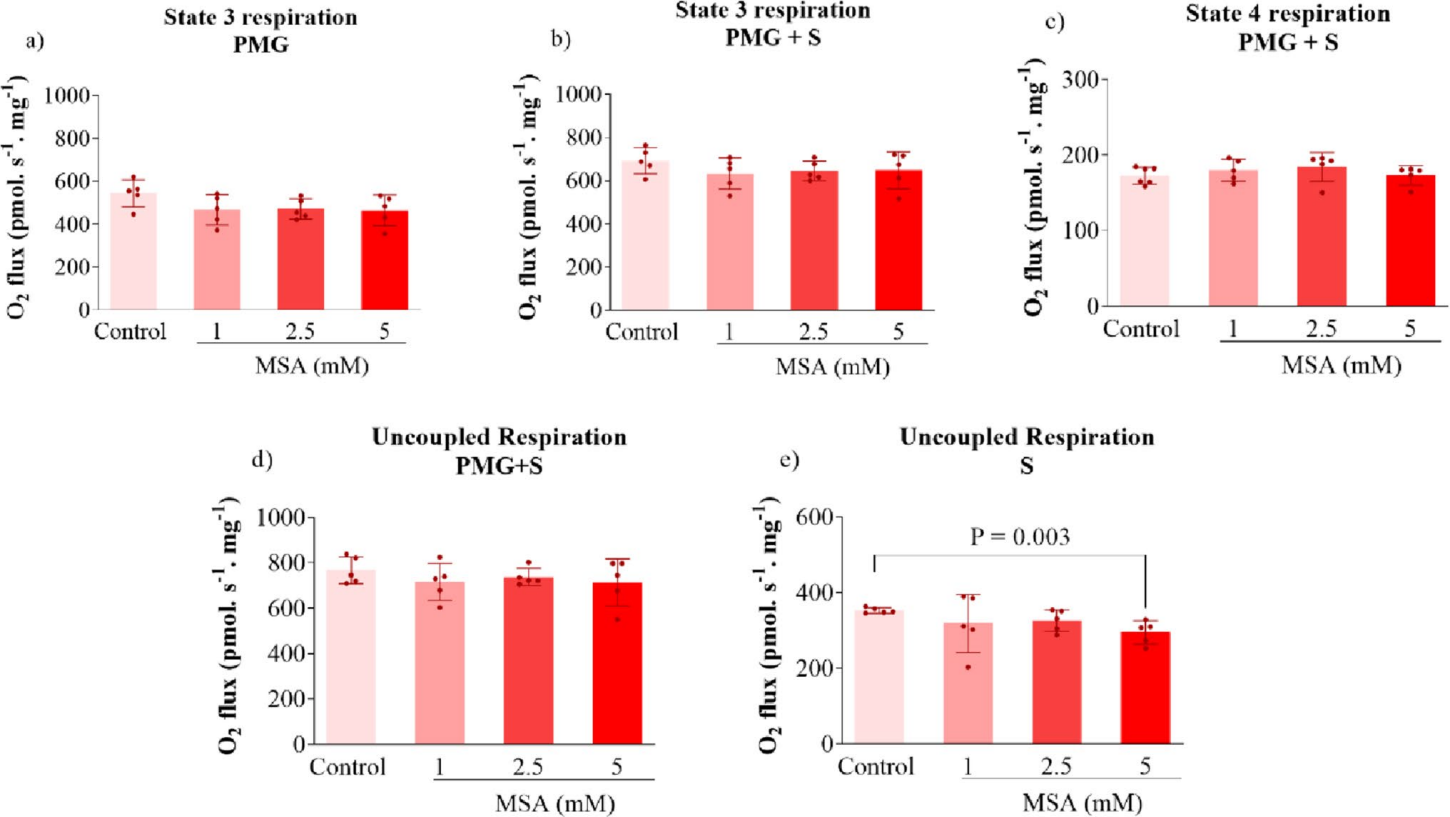
Effects of methylsuccinic acid (MSA) (1–5 mM) on mitochondrial respiration in rat striatum. State 3 respiration was measured in the presence of ADP (1 mM), and pyruvate (5 mM), malate (0.5 mM), and glutamate (10 mM) (PMG) (**a**) plus succinate (S; 10 mM) (**b**) as substrates to assess respiration linked to oxidative phosphorylation; state 4 was stimulated by oligomycin (ATP synthase inhibitor; 1 µg.mL^− 1^), to evaluate mitochondrial respiration not linked to ATP formation (resting respiration) (**c**). Uncoupled respiration was stimulated by CCCP (uncoupler; two pulses of 0.5 µM) (**d** and **e**). Values are mean ± standard deviation (*N* = 5). No significant differences were observed through Duncan’s multiple range test. P-values shown were obtained with Student’s *t*-test

Reports have shown that EE patients present with elevated lactate levels in the plasma and brain structures, including the basal ganglia [8, 48], which is indicative of a mitochondrial respiratory chain deficiency. Therefore, we evaluated the effects of EMA and MSA on mitochondrial respiration in mitochondrial fractions isolated from rat striatum. EMA significantly reduced state 3 of mitochondrial respiration in the presence of PMG (substrates linked to NADH) [*P* < 0.05] (Fig. 1a), as well as in the presence of PMG + S (substrates linked to NADH and FADH_2_) [*P* < 0.05] (Fig. 1b). Furthermore, EMA decreased uncoupled respiration stimulated only by succinate [*P* < 0.05] (Fig. 1e). In contrast, EMA did not change state 4 and uncoupled respiration driven by PMG + S (Fig. 1c and d). Additionally, we verified that MSA had a trend toward reduced uncoupled respiration with S (Figs. 2e). However, the other respiratory parameters were not affected by MSA (Fig. 2).

### EMA and MSA Impair CAC and ETC Functioning

**Fig. 3:**
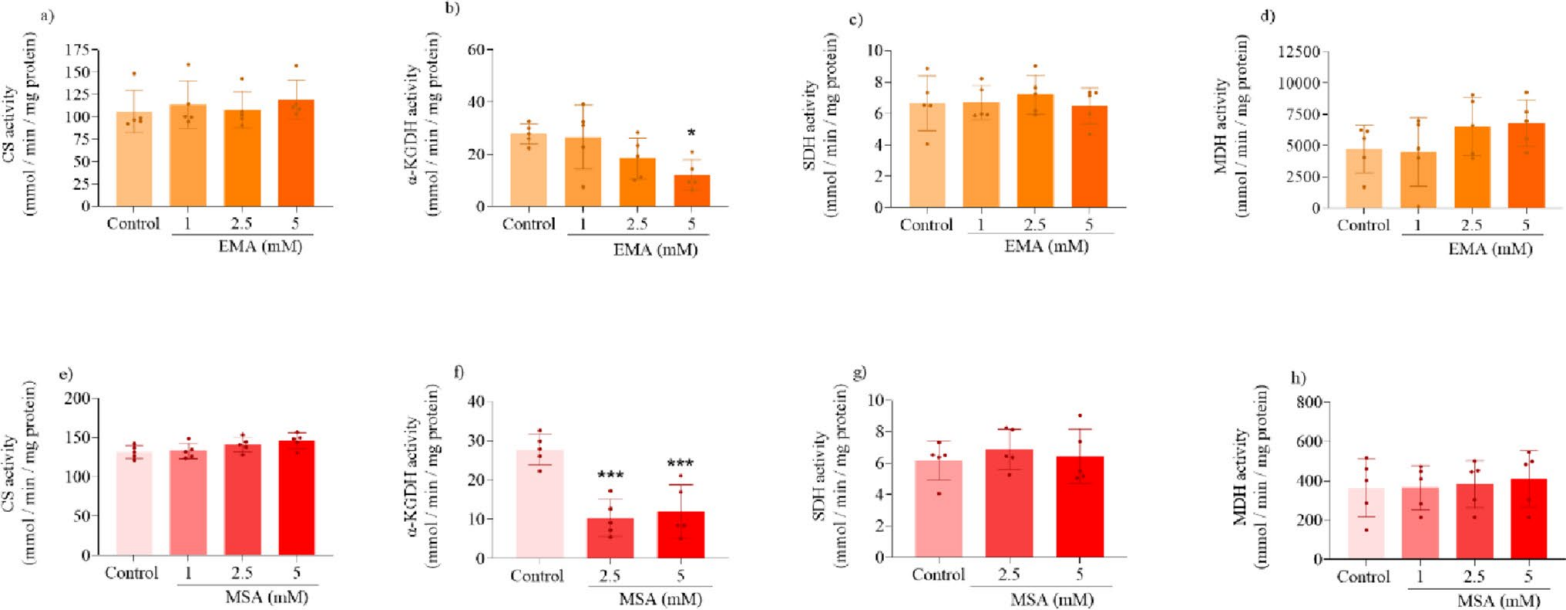
Effects of ethylmalonic acid (EMA) and methylsuccinic acid (MSA) (1–5 mM) on the activities of citric acid cycle enzymes in rat striatum. Activities of citrate synthase (CS) (**a** and **e**), α-ketoglutarate dehydrogenase (α-KGDH) (**b** and **f**), succinate dehydrogenase (SDH) (**c** and **g**) and malate dehydrogenase (MDH) (**d** and **h**) were measured. Values are mean ± standard deviation (*N* = 5). *P < 0.05, ***P < 0.001, compared to the control group (Duncan’s multiple range test)

**Fig. 4:**
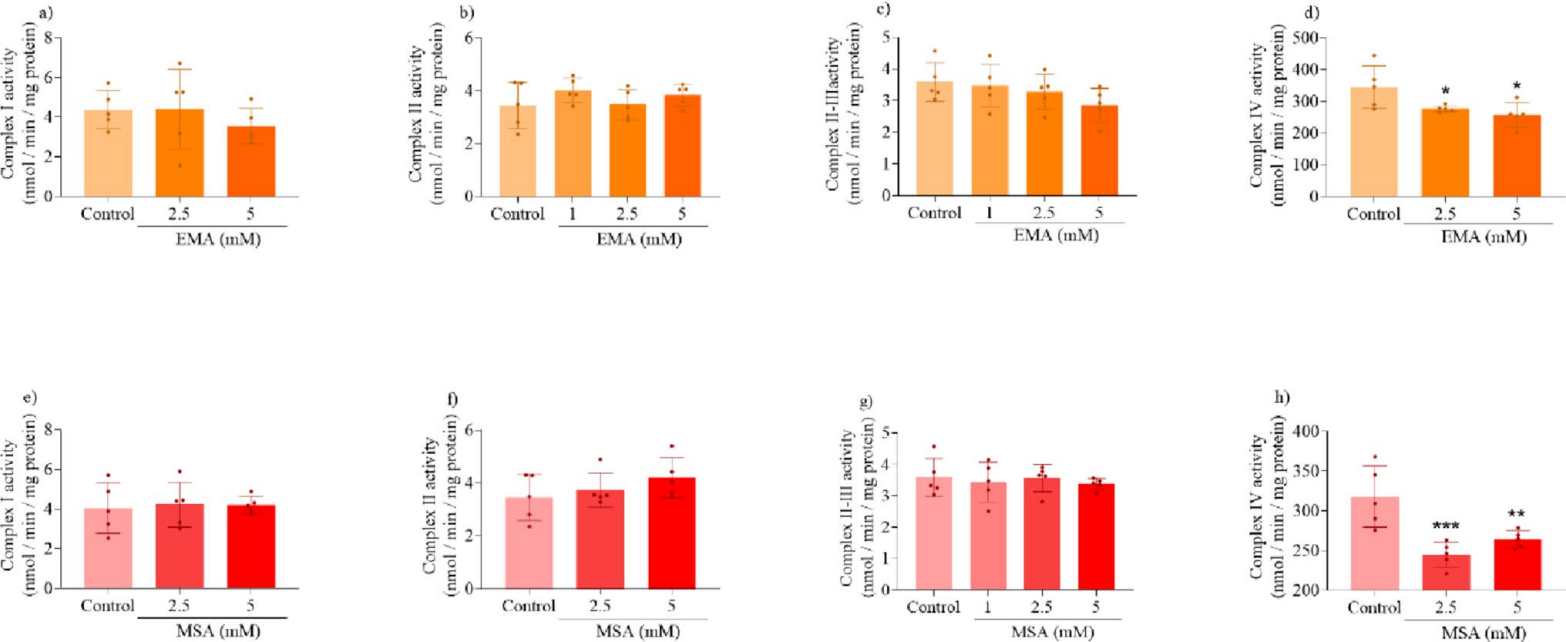
Effects of ethylmalonic acid (EMA) and methylsuccinic acid (1–5 mM) on the activities of electron transport chain complexes in rat striatum. Activities of complex I (**a** and **e**), complex II (**b** and **f**), complex II–III (**c** and **g**) and complex IV (**d** and **h**) were measured. Values are mean ± standard deviation (*N* = 5). *P < 0.05, **P < 0.01, ***P < 0.001, compared to the control group (Duncan’s multiple range test)

We also determined the effects of EMA and MSA on the activity of CAC enzymes and ETC complexes in striatal supernatants to investigate possible mechanisms underlying the mitochondrial respiration impairment. We found that both EMA and MSA markedly reduced α-KGDH activity, but did not change the activities of CS, MDH, and SDH (Fig. 3). Regarding ETC complexes, EMA and MSA significantly reduced the activity of complex IV in the striatum, with a higher inhibition caused by MSA, whereas the activities of complexes I, II and II-III were not altered by these metabolites (Fig. 4).

#### In silico Analysis of MSA Interaction with E1 and E3 α-KGDH Subunits and with Dicarboxylate, Glutamate, and α-Ketoglutarate/Malate Mitochondrial Carriers

**Fig. 5:**
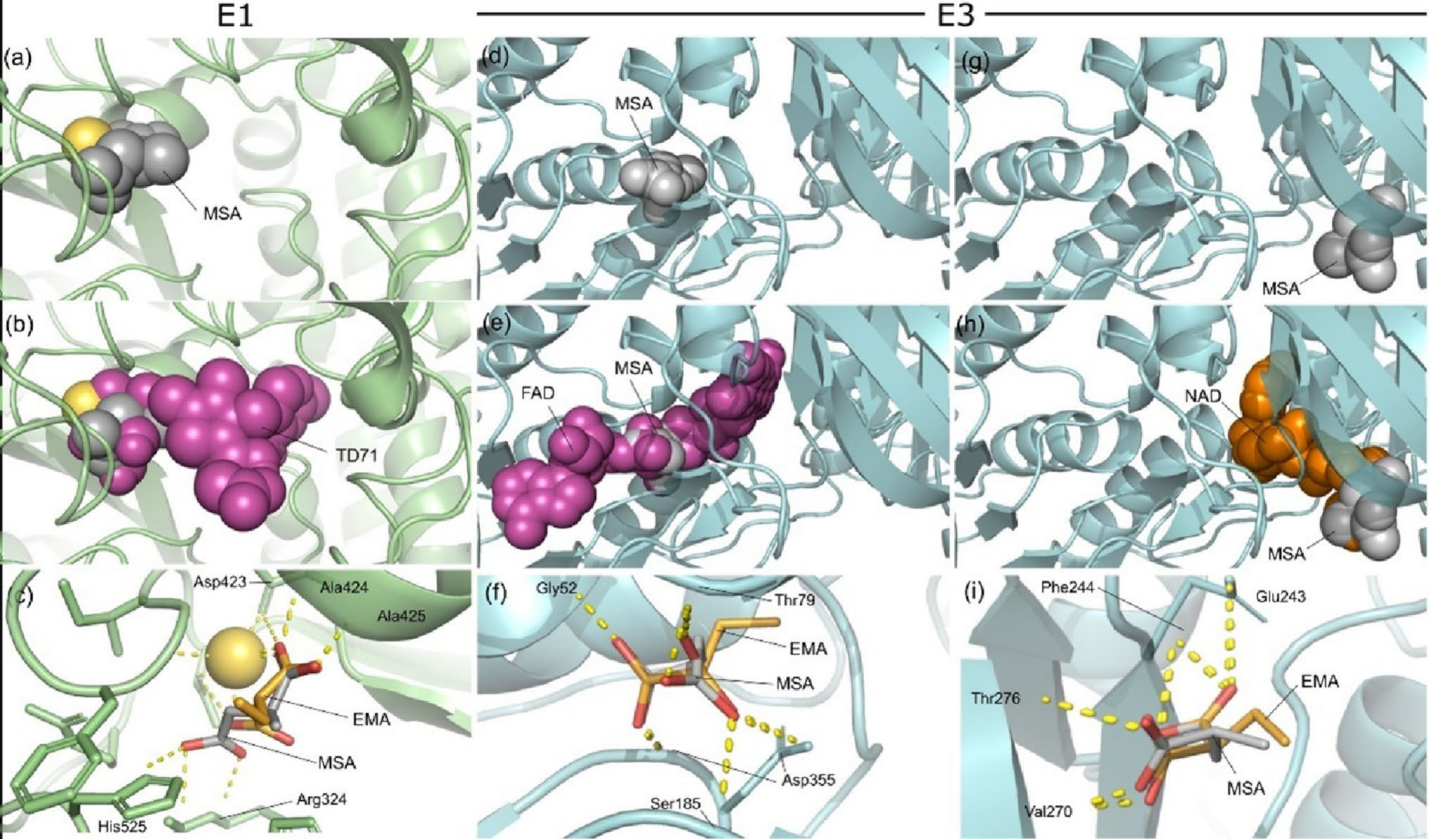
Cartoon representation of methylsuccinic acid (MSA) and ethylmalonic acid (EMA) binding mode in E1 (**a**–**c**) and E3 (**d**–**f**) subunits of pig α-ketoglutarate dehydrogenase. Panels a, d, and g show MSA in the catalytic, FAD and NAD^+^ binding sites; b, e and h show the overlay with TD71, FAD and NAD^+^; and c, f and i display the overlay between MSA and EMA at the respective binding sites

We previously demonstrated that EMA interacts with α-KGDH and competes with glutamate and succinate for their mitochondrial transporters [24]. As MSA is an EMA isomer and also inhibits the activity of α-KGDH, the same computational analysis was performed for this organic acid. It was observed that MSA accommodates in the active site of a Mg²⁺-dependent enzyme, overlapping with the reference inhibitor TD71 (Fig. 5a–c). This finding suggests that MSA can inhibit α-KGDH and reproduce the effect of compounds already characterized as negative modulators of these pathways [49–52]. In addition, we found that MSA occupies regions close to the cofactors FAD, NAD, and lipoic acid in the E3 subunit, suggesting that its presence may interfere with the catalytic dynamics of these molecules (Fig. 5d-i). Interestingly, it was observed that MSA may bind to α-KGDH sites with similar interaction strength as EMA (Table 1) [24].

**Fig. 6:**
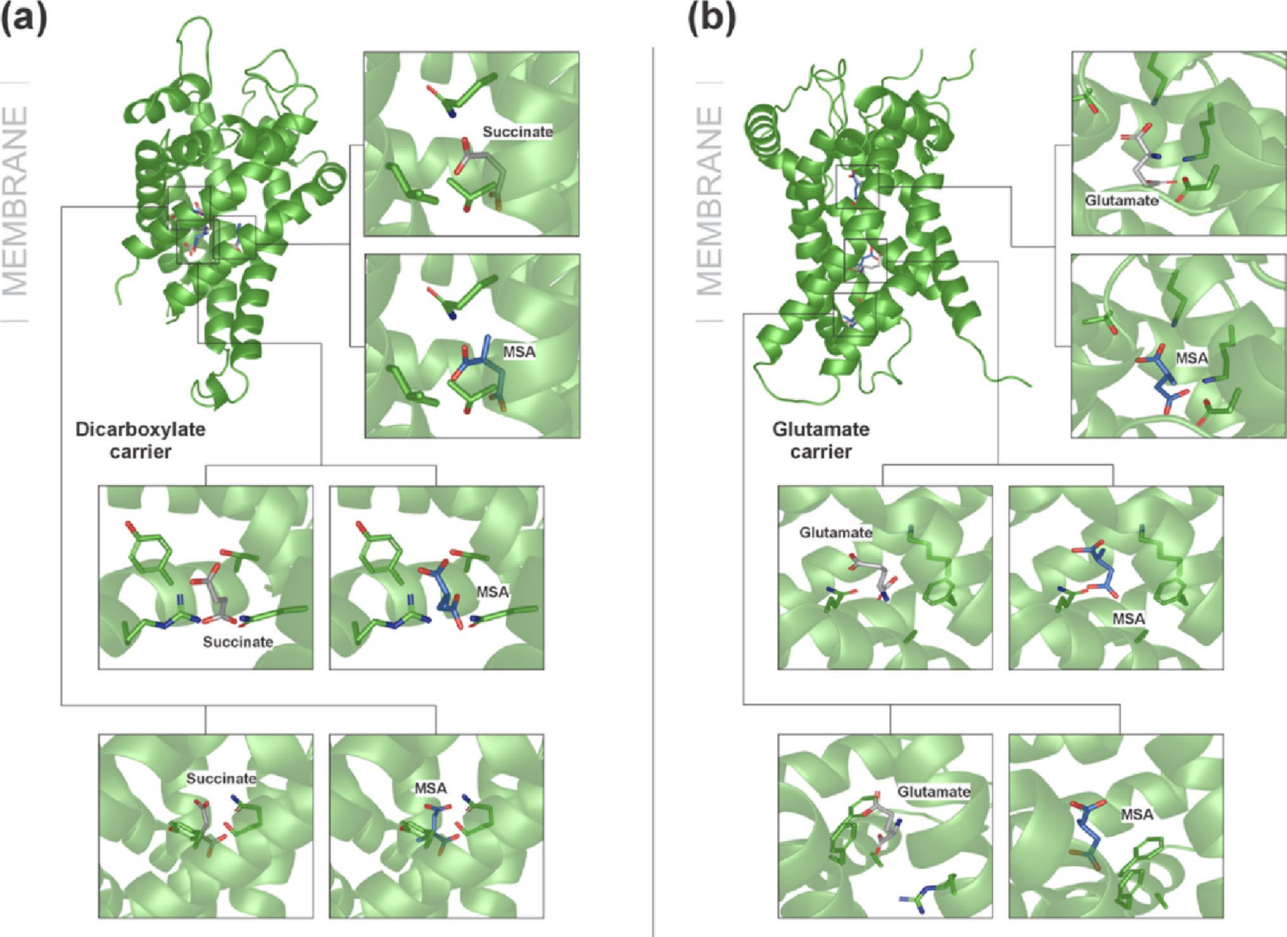
Cartoon representation of methylsuccinic acid (MSA) binding in the mitochondrial dicarboxylate (**a**) and glutamate (**b**) carriers

We also investigated the interaction of MSA with mitochondrial dicarboxylate and glutamate transporters. Docking simulations targeting these carriers showed that MSA has the potential to interact directly with these transporters. Figure 6 demonstrates that MSA may occupy the same binding sites as succinate (Fig. 6a) and glutamate (Fig. 6b), indicating that this metabolite can compete with physiological substrates, impairing both the supply of citric acid cycle intermediates and fundamental anaplerotic processes. The magnitude of all calculated binding forces obtained through docking simulations is shown in Table 1, comparing the MSA binding forces with those previously obtained for other ligands and EMA.

**Table 1:** Calculated docking energies of methylsuccinic acid (MSA) and ethylmalonic acid (EMA) binding in different subunits (E1 or E3) of pig α-ketoglutarate dehydrogenase and mitochondrial carriers

Enzyme site	Ligand	Energy (kcal/mol)
E1 – Cofactor binding site	enamine-ThDP^*^	− 7.7
	EMA^**^	− 5.2
	MSA	− 5.7
E3 – Cofactor binding site	FAD^*^	− 14.9
	EMA^**^	− 5.2
	MSA	− 5.3
E3 – Cofactor binding site	NADH^*^	− 9.7
	EMA	− 4.3
	MSA	− 4.4
E3 – Cofactor binding site	Lipoic acid^*^	− 5.4
(in the presence of FAD and NAD^+^)	EMA	− 4.9
	MSA	− 5.0
Mitochondrial dicarboxylate carrier	Succinate (site 1) ^*^	− 4.7
	Succinate (site 2) ^*^	− 4.6
	Succinate (site 3) ^*^	− 4.7
	EMA (site 1) ^**^	− 4.6
	EMA (site 2) ^**^	− 4.9
	EMA (site 3) ^**^	− 4.8
	MSA (site 1)	− 5.0
	MSA (site 2)	− 4.9
	MSA (site 3)	− 5.0
Mitochondrial glutamate carrier	Glutamate (site 1) ^*^	− 5.2
	Glutamate (site 2) ^*^	− 5.1
	Glutamate (site 3) ^*^	− 5.2
	EMA (site 1) ^**^	− 5.0
	EMA (site 2) ^**^	− 5.0
	EMA (site 3) ^**^	− 5.2
	MSA (site 1)	− 5.0
	MSA (site 2)	− 4.8
	MSA (site 3)	− 5.3
Mitochondrial α-ketoglutarate/malate carrier	α-ketoglutarate (site 1) ^*^	− 5.5
	α-ketoglutarate (site 2) ^*^	− 5.4
	EMA (site 1) ^**^	− 5.3
	EMA (site 2) ^**^	− 5.2
	MSA (site 1)	− 5.1
	MSA (site 2)	− 5.1

* Energy values were previously reported by Gamelon and Yoccoz [53]

** Energy values were previously reported by de Moura Alvorcem et al. [24]

### EMA and MSA Dissipate Mitochondrial Membrane Potential in Striatal Mitochondrial Fractions

**Fig. 7:**
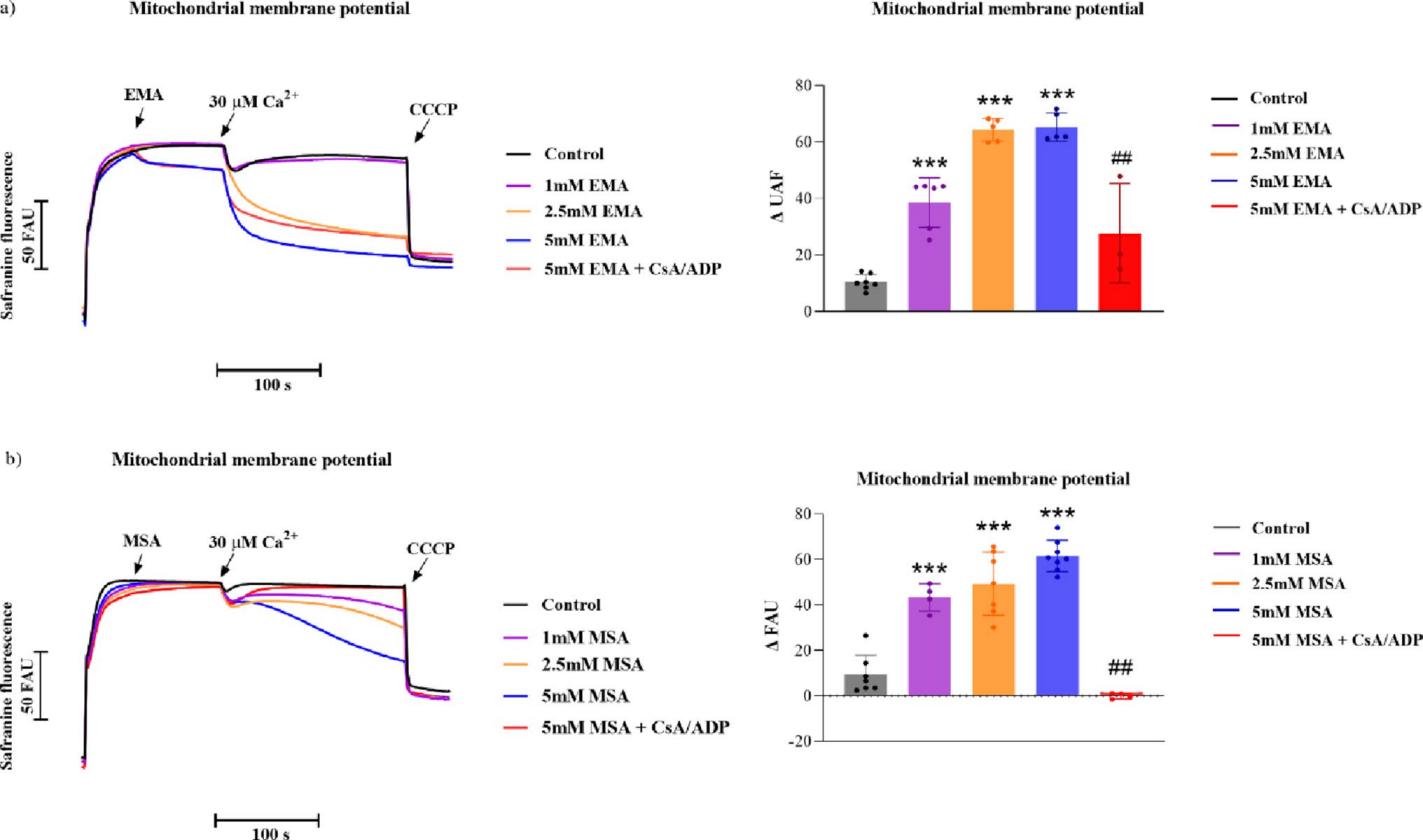
Dissipation of mitochondrial membrane potential (ΔΨm) by ethylmalonic (EMA; **a**) and methylsuccinic (MSA; **b**) acids in Ca^2+^-loaded striatal mitochondria using succinate as substrate. Experiments were performed in an incubation medium containing striatal mitochondrial preparations (0.75 mg protein.mL^− 1^), succinate (5 mM) and rotenone (0.5 µM). Cyclosporine A (CsA, 1 µM) and ADP (300 µM) were added at the beginning of the assays, and EMA (**a** and **b**) or MSA (**c** and **d**) (1–5 mM) 50 s later. All experiments refer to mitochondrial preparations supplemented with Ca^2+^ (30 µM), as indicated. CCCP (1 µM) was added at the end of the measurements to achieve maximal depolarization. Controls were performed in the absence of EMA and MSA. Values are mean ± standard deviation (*N* = 3–7). ***P < 0.001, compared to control groups; ##*P* < 0.01, compared to 5 mM EMA or MSA (Duncan’s multiple range test)

Next, we evaluated whether EMA and MSA (1–5 mM) could alter mitochondrial membrane potential in the striatum. We observed that both organic acids dissipated the membrane potential sustained by succinate in striatal mitochondria supplemented with (30 µM) Ca²⁺ (Figure 7a-EMA: *P* < 0.001; Fig. 7b-MSA: *P* < 0.001) (Fig. 7). Furthermore, the combination of cyclosporine A (CsA) and ADP, classical inhibitors of MPT, prevented the dissipation of mitochondrial membrane potential caused by both EMA (Fig. 7a) and MSA (Fig. 7b). These results suggest that both organic acids trigger the opening of the MPT pore.

## EMA Decreases Ca^2+^ Retention Capacity in Striatal Mitochondrial Fractions

**Fig. 8:**
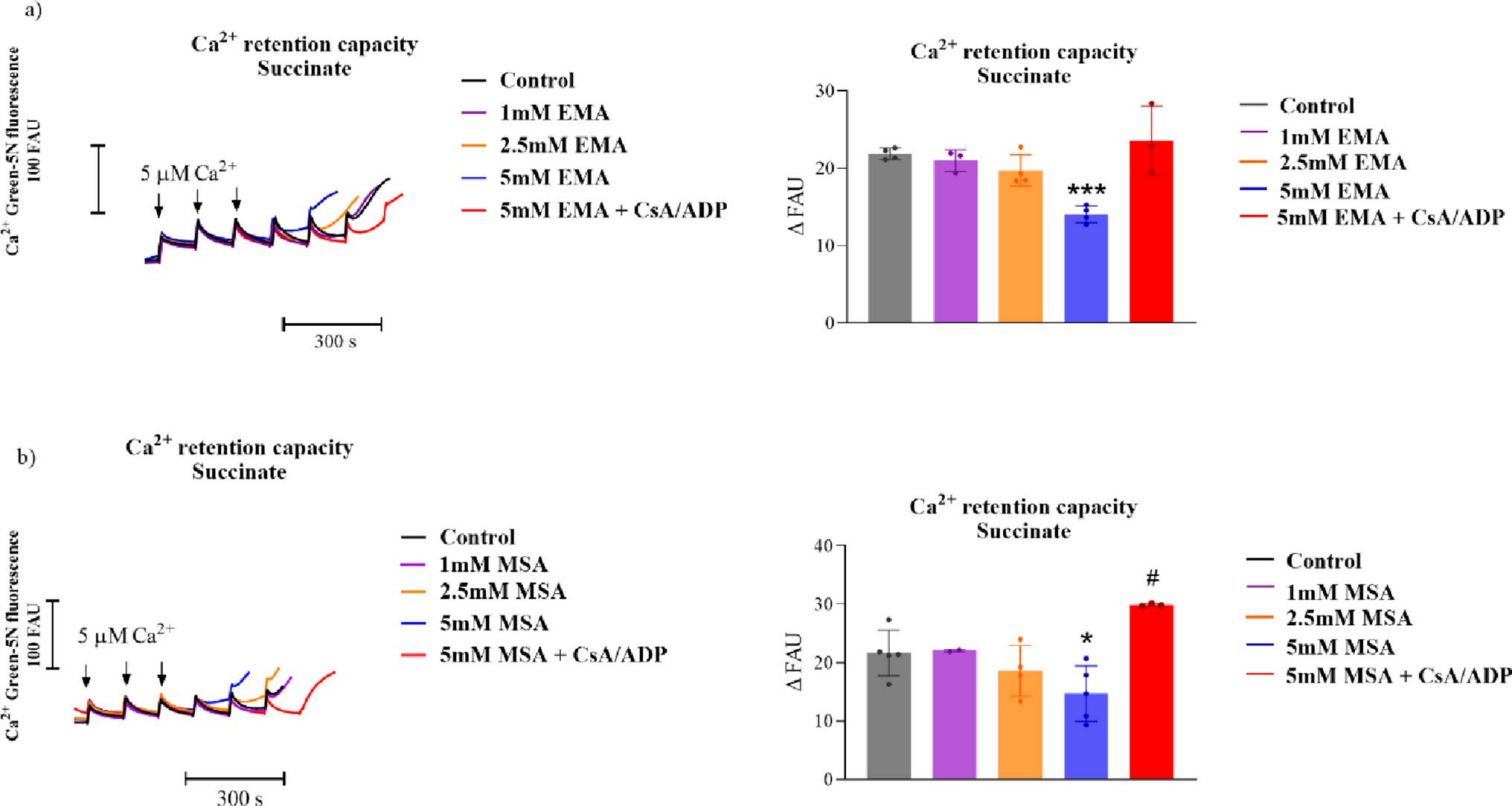
Reduction of Ca^2+^ retention capacity by ethylmalonic (EMA; **a**) and methylsuccinic (MSA; **b**) acids in Ca^2+^-loaded striatal mitochondria using succinate as substrate. Experiments were performed in an incubation medium containing mitochondrial preparations (0.75 mg protein.mL^− 1^), succinate (5 mM), and rotenone (0,5 µM). EMA or MSA (1–5 mM) and cyclosporin A (CsA, 1 µM) and ADP (30 µM) were added at the beginning of the assays. All experiments refer to mitochondrial preparations supplemented with sequential additions of 5 µM Ca^2+^, as indicated. CCCP (1 µM) was added at the end of the measurements to achieve maximal mitochondrial Ca^2+^ release. Controls were performed in the absence of EMA or MSA. Graphs show the quantification of the data. Values are mean ± standard deviation (*N* = 3–5) and are expressed as ΔFAU between 150 and 250 s. *P < 0.05, ***P < 0.001, compared to control groups; #*P* < 0.05, compared to 5 mM MSA (Duncan’s multiple range test)

Since mitochondrial Ca^2+^ homeostasis may be disrupted by induction of MPT [54], we investigated whether EMA and MSA could affect the Ca²⁺ buffering capacity of mitochondria. Figure 8 displays that the exposure to 5.0 mM EMA and MSA significantly reduced the Ca²⁺ retention capacity of succinate-sustained mitochondria, an effect that was prevented by the presence of CsA and ADP [Figure 7a-EMA: *P* < 0.001; Fig. 7b-MSA: *P* < 0.05] (Fig. 8). These data reinforce that EMA and MSA trigger MPT pore opening.

**Fig. 9:**
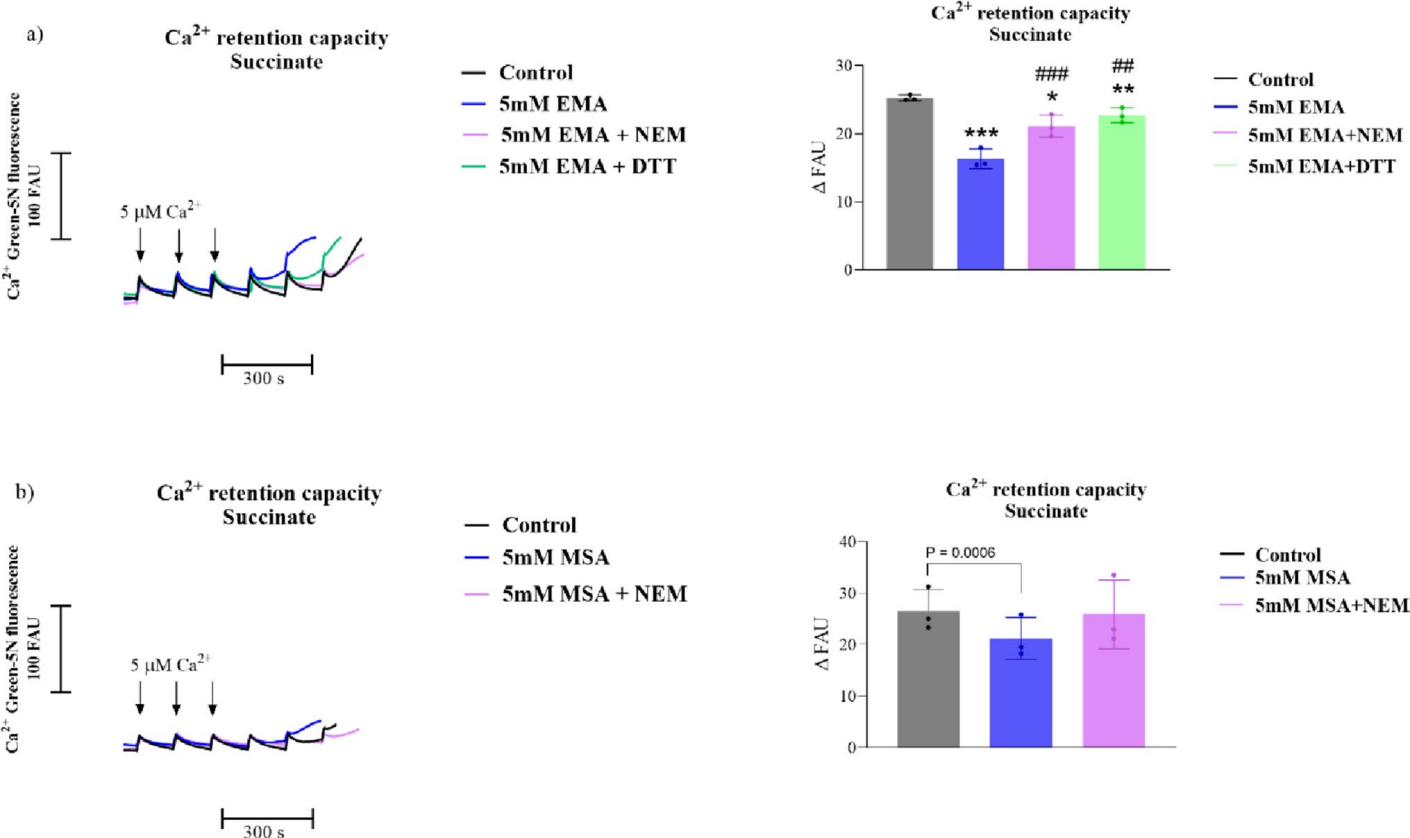
N-ethylmaleimide and dithiothreitol mitigate the reduction of Ca^2+^ retention capacity induced by ethylmalonic (EMA; **a**) and methylsuccinic (MSA; **b**) acids in Ca^2+^-loaded striatal mitochondria. Experiments were performed in an incubation medium containing mitochondrial preparations (0.75 mg protein.mL^− 1^), succinate (5 mM), and rotenone (0.5 µM). EMA or MSA (1–5 mM), N-ethylmaleimide (NEM; 10 µM), or dithiotreitol (DTT; 1,000 µM) were added at the beginning of the assays. All experiments refer to mitochondrial preparations supplemented with sequential additions of 5 µM Ca^2+^, as indicated. CCCP (1 µM) was added at the end of the measurements to achieve maximal mitochondrial Ca^2+^ release. Traces shown on the left are representatives and expressed as fluorescence arbitrary units (FAU). Panels on the right show the quantification of the data obtained after Ca^2+^ addition, respectively. Values are mean ± SD (*N* = 3). ***P < 0.001, compared to the control group; #*P* < 0.05, ###*P* < 0.001, compared to EMA (Duncan’s multiple range test). P-value shown in b was obtained with Student’s *t*-test

We next evaluated the influence of N-ethylmaleimide (NEM) and dithiothreitol (DTT) on the effects caused by EMA and MSA on Ca^2+^ retention capacity. Figure 9 demonstrates that EMA significantly decreased [*P* < 0.001], whereas MSA showed a strong trend [Student’s *t*-test: *P* < 0.001] to reduce Ca^2+^ retention capacity. Furthermore, NEM and DTT totally prevented the effect of EMA on this parameter and attenuated the effect induced by MSA. NEM or DTT per se did not cause significant changes in this parameter (Supplementary Fig. 1). These results indicate a role for oxidized components involved in this retention.

### EMA and MSA Compromise the Antioxidant Defense System in Striatal Supernatants

**Fig. 10:**
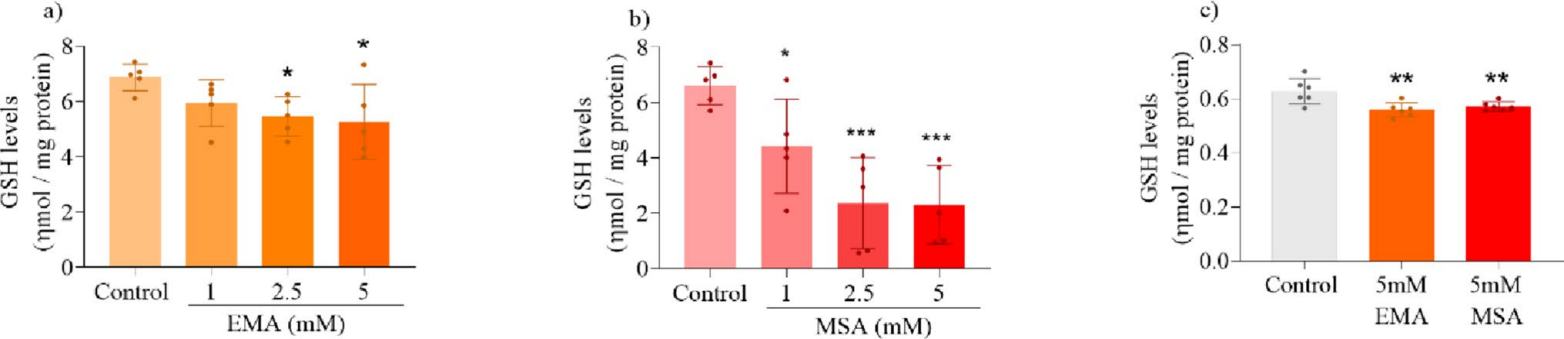
Effects of ethylmalonic acid (EMA) and methylsuccinic acid (MSA) (1-5mM) on reduced glutathione concentrations (GSH) in rat striatum supernatants (**a** and **b**) and mitochondrial fractions (**c**). Values are mean ± standard deviation (*N* = 5–6). *P < 0.05, **P < 0.01, ***P < 0.001, compared to the control group (Duncan’s multiple range test)

Since previous data showed that EMA causes oxidative stress in the brain of rodents [20] and MPT pore opening may be induced by elevated ROS levels [24], we investigated the effects of EMA and MSA on antioxidant defenses in rat striatum supernatants. Both EMA and MSA significantly decreased GSH levels (Fig. 10), with the highest effect caused by MSA, but in contrast, did not alter GPx and SOD activities (Supplementary Fig. 2). We also evaluated GSH levels in mitochondrial fractions and verified that both acids decreased this parameter (Fig. 10).

## Discussion

EE is often a fatal neurometabolic disorder characterized by severe neurological dysfunction associated with pronounced abnormalities in the basal ganglia and other brain structures [3, 12]. Previous studies have demonstrated that EMA, a main accumulating metabolite in EE, elicits toxic effects in the brain of rodents, indicating its relevant role in the pathophysiology of this disorder [21, 23, 55]. Thus, we investigated the effects of EMA on mitochondrial bioenergetics, as well as Ca²⁺ homeostasis and redox balance in the striatum of rats, a brain structure commonly affected in EE, that has not been previously studied. We also evaluated the effects of MSA, an EMA derivative that also accumulates in the disorder, whose effects remain unknown. EMA impaired state 3 of mitochondrial respiration driven by substrates related to NADH (PMG) and FADH₂ (PMG + S) electron transfer. Furthermore, both organic acids disturbed uncoupled respiration, suggesting a direct inhibitory effect on mitochondrial metabolism as the resting state is predominantly regulated by proton leak and only approximately 10% by substrate oxidation [56]. Moreover, EMA and MSA inhibited α-KGDH activity. In agreement with this, we previously found that EMA impaired mitochondrial respiration and reduced α-KGDH activity in rat cerebellum [24], indicating that the inhibition of this enzyme may underlie mitochondrial respiration impairment across different brain structures. In this context, α-KGDH is widely recognized as a crucial control step in the CAC, and its inhibition is likely to impair NADH production [57].

Other novel findings of the present investigation using docking simulations revealed that MSA can bind to multiple sites on α-KGDH, including its active site and regions near the binding sites for FAD, NAD, and lipoic acid in the E3 subunit. MSA can also bind at the same site as succinate inside the pore of the mitochondrial dicarboxylate and as glutamate in the glutamate carrier, indicating that MSA impairs the mitochondrial transport of these substrates. Interestingly, we recently showed that EMA can also bind to α-KGDH as well as to mitochondrial dicarboxylate and glutamate carriers [24]. We further demonstrated, for the first time, that EMA and MSA inhibited complex IV activity, a primary regulation point in the ETC [58]. Therefore, our findings show that EMA and MSA impair striatal bioenergetics through similar mechanisms.

Next, we verified that both acids dissipated the mitochondrial membrane potential sustained by succinate in striatal mitochondria incubated with Ca²⁺. Noteworthy, disturbances in mitochondrial membrane potential are particularly critical to the striatum as this brain structure has high energy demands and is highly vulnerable to mitochondrial dysfunction [59]. EMA and MSA also reduced Ca^2+^ retention capacity, which is consistent with the loss of mitochondrial membrane potential caused by these acids, since the electrochemical gradient is essential for the uptake of this ion [60].

Experimental evidence shows that CsA can preserve mitochondrial membrane potential, even in cases of intracellular Ca^2+^ accumulation, by blocking cyclophilin D and consequently halting the occurrence of MPT [61]. ADP has also been reported as a potent inhibitor of MPT in isolated brain organelles through its binding to the adenine nucleotide translocator [43]. We found that the combination of CsA and ADP prevented the dissipation of membrane potential and the decrease in Ca²⁺ retention capacity induced by EMA and MSA. These data suggest that the synergism between EMA or MSA and Ca²⁺ can modulate mitochondrial permeability and induce the opening of MPT pore, accelerating membrane potential loss and the release of calcium. Interestingly, it was recently reported that MPT underlies axonal injury caused by intracerebral hemorrhage induction in the striatum of mice and that its prevention ameliorated corticospinal tract injury and motor dysfunction [62]. Blockage of MPT also improved the motor deficit and prevented the loss of nigral dopaminergic neurons projecting into the striatum in a rat model for Parkinson’s disease [63]. Thus, we propose that MPT induction is an important mechanism involved in the striatal neuronal damage observed in EE.

We sought to investigate strategies capable of preventing mitochondrial collapse and protecting the organelle against the toxic effects of EMA and MSA. To this end, we evaluated the effects of NEM and DTT, agents known for their ability to preserve the mitochondrial membrane potential and interrupt events that may lead to mitochondrial failure. While NEM is an alkylating compound that reacts with free sulfhydryl groups of pore proteins, preventing covalent modifications that may trigger pore opening [64], DTT acts as a reducing agent, restoring disulfide bonds and stabilizing mitochondrial proteins [65]. NEM and DTT prevented the effects provoked by both EMA and MSA, indicating that the modulation of the redox state of mitochondrial proteins prevents the opening of MPT pore by these acids.

Given that MPT pore opening may be modulated by reactive species [66–68], we assessed parameters related to oxidative stress. EMA and MSA caused a significant decrease in GSH levels, indicating a compromise of striatal antioxidant defenses, favoring oxidative damage and neurochemical dysfunction [69]. In this particular, the striatum is a brain structure known for its intense dopaminergic activity and vulnerability to oxidative stress [69]. Moreover, this finding is consistent with our hypothesis that EMA and MSA alter critical thiol groups that regulate the MPT pore opening [67]. Regarding the activities of SOD and GPx, since our supernatants lacked nuclei, we assessed only the direct effects of EMA and MSA on the protein structures of these enzymes. We cannot rule out that EMA and MSA may influence the gene expression of these enzymes.

It is challenging to determine the pathophysiological significance of our findings because our model has some limitations. To our knowledge, there are no reports on the concentrations of EMA and MSA in the brains of patients with EE. However, it is widely accepted that, in the so-called cerebral organic acidurias, the production and trapping of acidic compounds in neural cells, particularly dicarboxylates and acyl-CoA ester precursors, lead to their accumulation in the brain, causing toxic effects and progressive neurodegeneration [17]. Additionally, it is probable that episodes of metabolic decompensation in EE patients cause significant increases in brain levels of EMA and MSA due to accelerated catabolism. Therefore, we believe that the toxic effects induced by these organic acids, as shown here, contribute to the pathophysiology of EE.

## Conclusion

Our data demonstrate that EMA and MSA impair mitochondrial bioenergetics by inhibiting α-KGDH and complex IV activities and induce MPT in the striatal tissue. We also reveal that EMA- and MSA-induced MPT is mediated by redox changes in cysteinyl groups of the permeability transition pore proteins. It is also important to note that our results are consistent with previous data demonstrating impaired mitochondrial respiration in fibroblasts from patients [70]. In the patient context, we suggest that these alterations may contribute to the lactic acidosis frequently described in patients with EE [8, 71]. Further research in patient-derived neural cells should be performed to confirm whether EMA and MSA induce MPT pore opening, potentially leading to cell death.

## Supplementary Information

Below is the link to the electronic supplementary material.

Supplementary Material 1

## Data Availability

No datasets were generated or analysed during the current study.
