# Supplementary material for: Ethylmalonic and Methylsuccinic Acids Disrupt Bioenergetics and Induce Mitochondrial Permeability Transition Through Thiol Redox Modulation in Rat Striatum: Potential Mechanisms Involved in Ethylmalonic Encephalopathy
Source: Neurochem Res. 2026 May 27;51(3):176. doi: 10.1007/s11064-026-04801-y (PMC13216122; doi:10.1007/s11064-026-04801-y)
Supplement: Supplementary file 1 — Supplementary Material 1 [file 11064_2026_4801_MOESM1_ESM.docx]

**Ethylmalonic and methylsuccinic acids disrupt bioenergetics and induce mitochondrial permeability transition through thiol redox modulation in rat striatum: potential mechanisms involved in ethylmalonic encephalopathy**

Manuela Bianchin Marcuzzo^1^, Ângela Beatris Zemniaçak^1^, Jaqueline Santana da Rosa^1^, Bianca Silveira Signorini Verdi^1^, Maria Paula Dalla Vechia Benati^1^, Josyane de Andrade Silveira^1^, Juliana Gomes do Nascimento^1^, Helena de Almeida Moreira^1^, Mateus Dias-Oliveira^2^, Diogo Onofre Souza^1^, Alexandre Umpierrez Amaral^1,3^, Geancarlo Zanatta^2^, Moacir Wajner^1,4^, Guilhian Leipnitz^1,5,6,7,8^

^1^Programa de Pós-Graduação em Ciências Biológicas: Bioquímica, Instituto de Ciências Básicas da Saúde, Universidade Federal do Rio Grande do Sul, 90035-003 Porto Alegre, RS, Brazil

^2^Postgraduate Programme in Cellular and Molecular Biology (PPGBCM), Center of Biotechnology, Federal University of Rio Grande do Sul, 90650-001 Porto Alegre, RS, Brazil

^3^Programa de Pós-Graduação em Atenção Integral à Saúde (UNICRUZ/URI-Erechim/UNIJUÍ), Universidade Regional Integrada do Alto Uruguai e das Missões, Erechim, Rio Grande do Sul, Brazil

^4^Serviço de Genética Médica, Hospital de Clínicas de Porto Alegre, Porto Alegre, Brazil

^5^Departamento de Bioquímica, Instituto de Ciências Básicas da Saúde, Universidade Federal do Rio Grande do Sul, 90035-003 Porto Alegre, RS, Brazil

^6^Programa de Pós-Graduação em Ciências Biológicas: Neurociências, Instituto de Ciências Básicas da Saúde, Universidade Federal do Rio Grande do Sul, 90035-003 Porto Alegre, RS, Brazil

^7^Programa de Pós-Graduação em Ciências Biológicas: Fisiologia, Instituto de Ciências Básicas da Saúde, Universidade Federal do Rio Grande do Sul, 90035-003 Porto Alegre, RS, Brazil

^8^Functional Pharmacology and Neuroscience, Department of Surgical Sciences, Uppsala University, 75124, Uppsala, Sweden

^*^ Corresponding Author: Guilhian Leipnitz, Department of Biochemistry, Institute of Basic Health Sciences, Universidade Federal do Rio Grande do Sul, Porto Alegre, RS 90035-003, Brazil. Phone: +55 51 3308-5571, e-mail: [guilhian@ufrgs.br](mailto:guilhian@ufrgs.br)


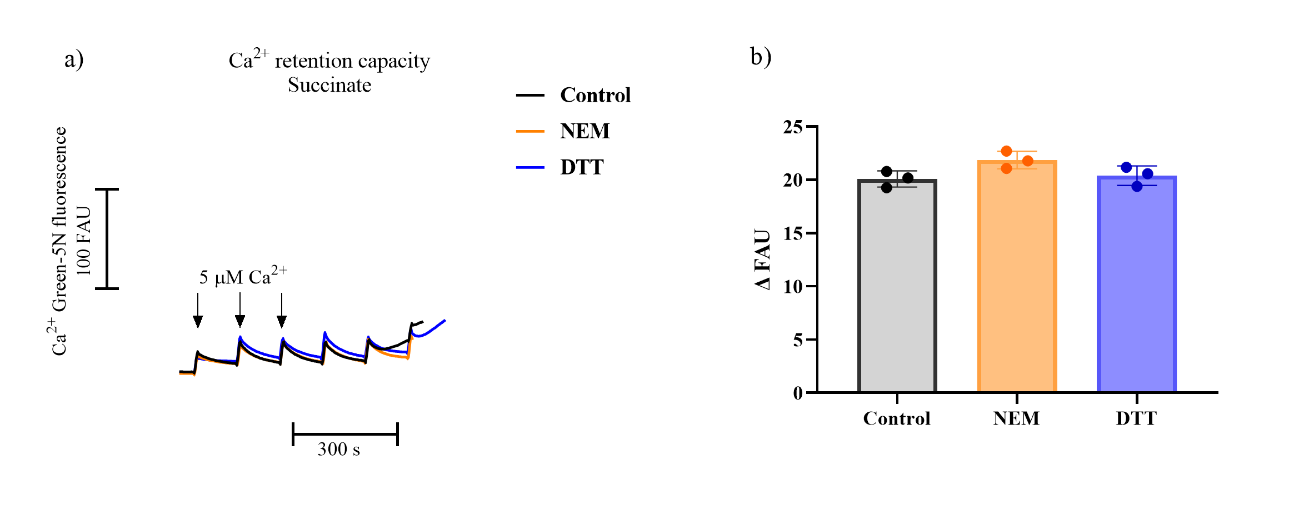


**Supplementary Figure 1:** Ca^2+^ retention capacity by N-ethylmaleimide (NEM) and dithiothreitol (DTT) in Ca^2+^-loaded striatal mitochondria using succinate as substrate. Experiments were performed in an incubation medium containing mitochondrial preparations (0.75 mg protein.mL^-1^), succinate (5 mM), and rotenone (0.5 μM). NEM (10 μM), or DTT (1000 μM) was added at the beginning of the assays. All experiments refer to mitochondrial preparations supplemented with sequential additions of 5 μM Ca^2+^, as indicated. CCCP (1 μM) was added at the end of the measurements to achieve maximal mitochondrial Ca^2+^ release. Controls were performed in the absence of NEM or DTT. Graphs show the quantification of the data. Values are mean ± standard deviation (N=3) and are expressed as ΔFAU between 150 and 250 s. No significant alterations were observed (Duncan's multiple range test).


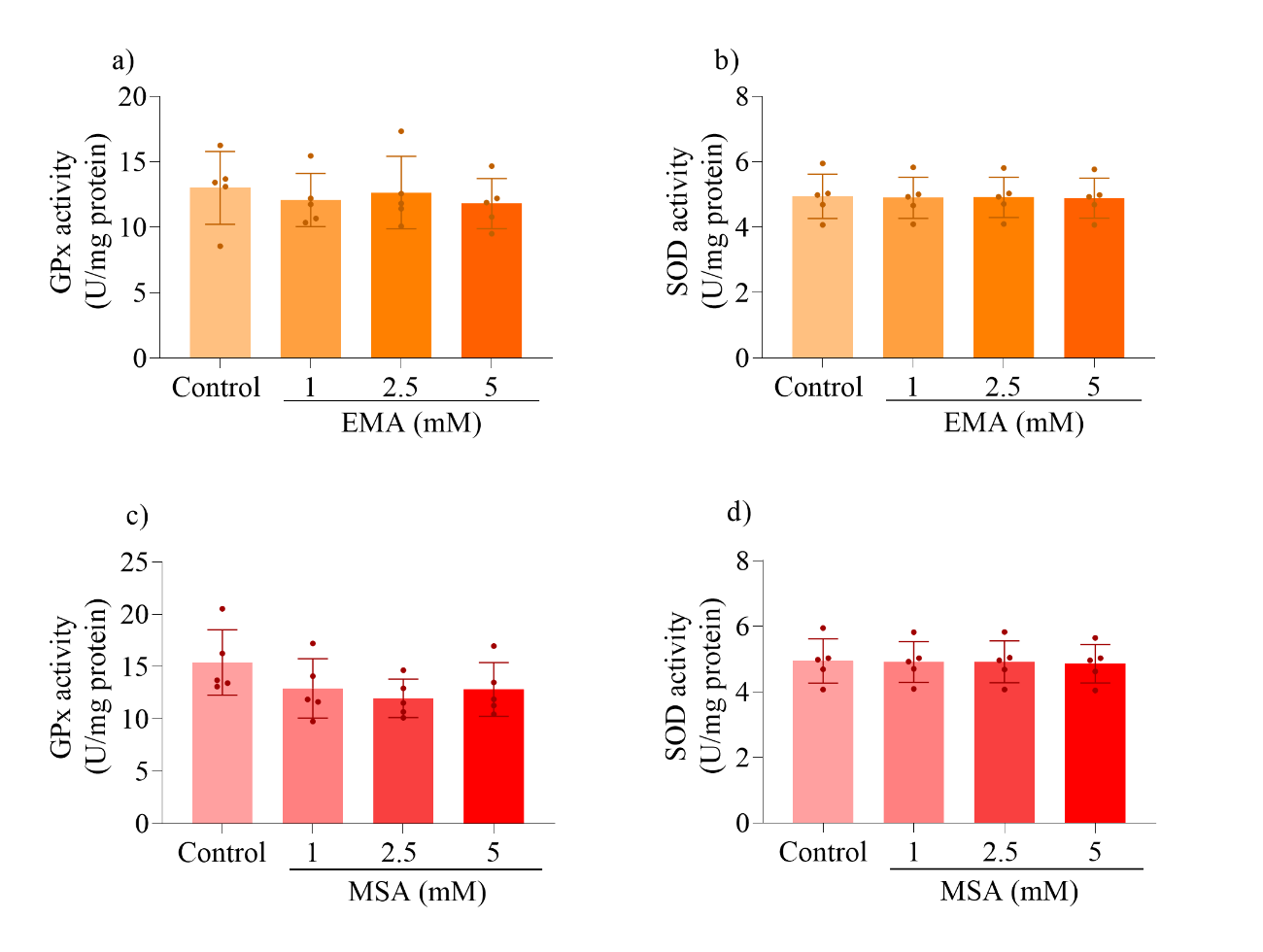


**Supplementary Figure 2:** Effects of ethylmalonic acid (EMA) and methylsuccinic acid (MSA) (1-5 mM) on the activities of glutathione peroxidase (GPx) (a and c) and superoxide dismutase (SOD) (b and d) in rat striatum. Values ​​are mean ± standard deviation (N=5). No significant alterations were observed (Duncan's multiple range test).
